# Encrusted Ureteral Stent Retrieval Using Flexible Ureteroscopy with a Ho: YAG Laser

**DOI:** 10.1155/2012/862539

**Published:** 2012-04-03

**Authors:** Takashi Kawahara, Hiroki Ito, Hideyuki Terao, Takehiko Ogawa, Hiroji Uemura, Yoshinobu Kubota, Junichi Matsuzaki

**Affiliations:** ^1^Department of Urology, Ohguchi Higashi General Hospital, 2-19-1 Irie, Kanagawa-ku, Yokohama City, Japan; ^2^Department of Urology, Graduate School of Medicine, Yokohama City University, Yokohma city, Japan

## Abstract

A 23-year-old female had bilateral ureteral stents placed due to bilateral renal stones and hydronephrosis. The bilateral ureteral stents were changed every 3 months. A kidney ureter bladder (KUB) film showed left encrustation along the ureteral stent thus necessitating removal; however, the ureteral stent could not be removed cystoscopically. The ureteral stent was, therefore, extracted using flexible ureteroscopy (URS) with a holmium (Ho): yttrium aluminum garnet (YAG) laser.

## 1. Introduction

In 1967, Zimskind et al. first reported the use of silicone ureteral splints to cystoscopically relieve ureteral obstructions [1]. Ureteral stents have since become a fundamental part of many urological procedures, including the treatment of obstructing ureteral calculi, ureteral stricture or ureteropelvic junction obstructions, retroperitoneal tumors or fibrosis, or after either open or endoscopic ureteral surgery [1, 2].

Serious complications still occur, including migration, fragmentation, and stone formation, especially when stents have been in place for a long time [2–7]. However, there are no widespread guidelines for the management of these potentially serious problems [8].

Shock wave lithotomy (SWL) and ureteroscopy (URS) are the first step in the removal encrusted ureteral stent. This report presents a case of successful encrusted ureteral stent removal by means of flexible URS with a holmium (Ho): yttrium aluminum garnet (YAG) laser.

## 2. Case Report

A 23-year-old female with bilateral renal stones presented with urosepsis and hydronephrosis. She underwent ureteral stenting for the bilateral ureters. Her previous history included anorexia nervosa since 22 years of age. She had no particular family history. Her personal history revealed the abuse of a purgative medicine for the purpose of losing her weight.

**Figure 1:**
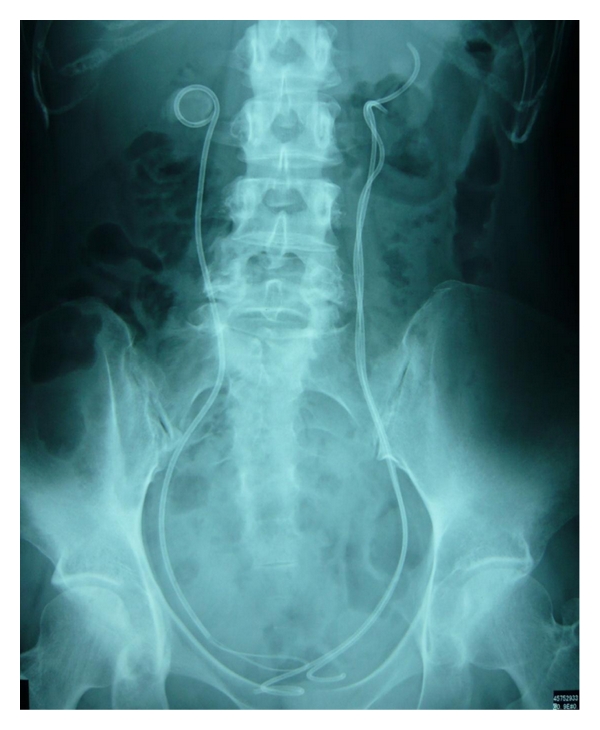
KUB shows bilateral renal stones. The newer stent was inserted beside the encrusted ureteral stent.

 The patient noticed right flank pain in May 2009 and was referred to the hospital. A kidney-urinary-bladder (KUB) film and computed tomography (CT) revealed bilateral renal and ureteral stones and bilateral hydronephrosis, and bilateral ureteral stents were inserted. She was not treated for the renal stones because her anorexia nervosa was uncontrollable and she sometimes experienced cardiac arrest due to an electrolyte abnormality. As a result, it was determined that she should be admitted to the Department of Psychiatry. The bilateral ureteral stents were exchanged within three months (from 2 weeks to 3 months). The left polyurethane ureteral stent (Polaris Loop, Boston Scientific, MA, USA) could not be removed because of heavy encrustation in April 2001. Another ureteral stent was inserted in addition to the encrusted ureteral stent (Figure 1). SWL is one of the recommended procedures for removing an encrusted ureteral stent. However, since the patient's mental condition was unstable and the ureteral stent's encrustation was severe, we, therefore, chose to surgically remove the encrusted ureteral stent.

 A physical examination revealed emaciation with a body weight of 32 kg in body height 161 cm and no other abnormal physical findings. A laboratory examination showed decreased Hb (11.9 g/dL), increased Plt (502,000/*μ*L), and decreased K (2.3 mEq/L). Urinalysis showed RBC: >100/hpf and WBC >100/hpf, and urinary culture showed *Pseudomonas aeruginosa*.

**Figure 2:**
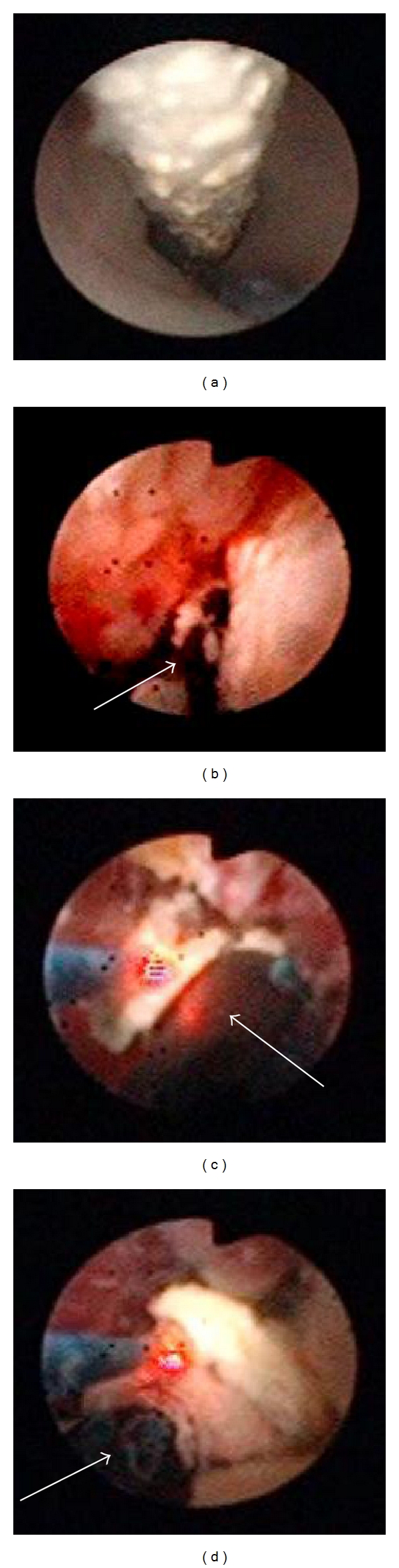
(a) The body of the encrusted ureteral stent. (b) Flexible URS shows that the upper end of the encrusted ureteral stent was heavily calcified to the renal stone. (c), (d) Lithotripsy around the encrusted stent using Ho: YAG laser (ureteral stent: arrow).

The patient's low potassium level became life-threatening in June 2010, so she was transferred to our department from the psychiatric hospital. The newer stent was removed cystoscopically under general anesthesia in the lithotomy position. Ceftazidime hydrate was given both preoperatively and postoperatively. The newer stent was slightly encrusted; however, it could be easily removed. The distal end of old encrusted stent was extracted from the external urethral orifice using foreign body forceps and controlled by mosquito forceps. A 6Fr rigid URS was inserted to the ureter beside the encrusted ureteral stent toward to the renal pelvis under guide wire assistance in order to observe the ureter and ureteral stent (Figure 2(a)). The ureteral stent was slightly encrusted but did not adhere to the ureter in the middle of the stent. However, a ureteral stent was found to be heavily encrusted at the proximal end of the loop in the renal collecting system. As a result, the stent could not be successfully approached using rigid URS. A 12 Fr ureteral access sheath (UAS) (Flexor, COOK Urological, USA) was inserted into the ureter beside the ureteral stent to observe the renal pelvis using a 5.3Fr flexible URS (URF-P5, Olympus, Japan) to determine the association of the ureteral stent and renal stone (Figure 2(b)). Intracorporeal lithotripsy was performed to control the ureteral stent, around the encrusted stent using the Ho: YAG laser (using a 200 *μ*m fiber, 1.0 J, 5 Hz; Versa Pulse 30 W, LUMENIS surgical, USA; Figures 2(c) and 2(d)).

The stent was carefully removed under fluoroscopic guidance. The total operation time was 65 minutes. An 8 Fr 24 cm polyurethane ureteral stent (Inlay Optima, BARD, NJ, USA) was inserted after URS. The retrieved ureteral stent was completely black and heavily encrusted at the proximal end of the loop. An analysis of the encrusting material showed the presence of ammonium acid urate urinary calculi. Since then, her medical condition has become controllable, and she has been stone free in her right kidney as of October 2011.

## 3. Discussion

Ureteral stents were first developed in 1967. Various materials and coatings have been developed to avoid ureteral stent complications such as encrustation, incrustation, and infections [1]. The incidence of encrustation increases with the duration that the stent remains in place [9]. Therefore, stents require periodic replacement or removal. A report by El-Faqih et al. indicated that stent encrustation rate increases from 9.2% for an indwelling time of less than 6 weeks to 47.5% at 6 to 12 weeks to 76.3% at more than 12 weeks [9]. And our previous reports also support that data [6]. Although the duration of time until a stent becomes heavily encrusted is generally considered to be approximately three months, we recommend removing ureteral stents as soon as possible to avoid potential encrustation [6, 10].

Bultitude et al. reported that 42.8% of the stents become difficult to remove cystoscopically within 4 months and 14.3% at 2 months [11, 12]. Okuda et al. reported on 15 irremovable ureteral stents in Japanese patients. The mean indwelling time of these stents was 20 months [13].

The development of the Ho: YAG laser revolutionized the treatment of urolithiasis. Thermal drilling is precise (0.5 mm) and powerful enough to vaporize any type of calculus [14]. Ho: YAG lithotripsy has the advantages of inducing both less soft tissue injury and less bleeding [14]. URS with a Ho: YAG laser is suitable for removal of heavily encrusted ureteral stents with impacted stones [11]. Kural et al. reported the removal of a UroLume using a Ho: YAG laser [15]. Bedke et al. described the fragmentation process of the ureteral stent using a Ho: YAG laser *in vitro* [16]. In the present case, we made stone fragments from the encrusted ureteral stent, taking care not to cut off the encrusted stent. Stents that cannot be removed with SWL and URS require PCNL, and open surgery should be performed [2, 11, 17–20]. Bultitude et al. reported that 27 of 38 encrusted stents (71.1%) could be removed using flexible/rigid URS in a single procedure [12]. The development of the flexible URS and Ho: YAG laser allows for both the observation and manipulation of stents in the renal pelvis [12, 17]. In some previous reports, extracting a ureteral stent using rigid URS was useful for the removal proximal encrustation. However, the extraction of a ureteral stent always has a risk of the ureter being cut by the stent during the removal process. Therefore, choosing flexible URS with UAS should be determined only after checking to make sure that there is no adherence of the stent to the ureter in the middle ureter [13, 18]. In a previous study, no ureteral stricture was reported after removing the ureteral stent; however, UAS is associated with the potential risk of ureteral stricture. Therefore, we inserted a ureteral stent at the conclusion of the ureteral stent retrieval.

A ureteral stent was inserted beside the encrusted ureteral stent for drainage and dilation of the ureter in the current case. UAS can facilitate URS and the retrieval of stones fragments while reducing the intrarenal pressure, thereby improving irrigate flow and decreasing operative time [21, 22]. The ureteral stent is also useful for passive dilation of the ureter in children [23, 24]. UAS is effective for intrarenal lithotripsy to treat an upper encrusted ureteral stent. Therefore, inserting a ureteral stent beside an encrusted ureteral stent before URS is thought to be useful. In our case, we intended to insert a new ureteral stent beside the encrusted ureteral stent. In the previous reports, preoperative stenting facilitated the dilation of the ureter and also resulted in the insertion of a large caliber UAS [25, 26]. This preoperative stenting procedure facilitated the insertion of the UAS beside the encrusted ureteral stent.

This paper presented a case where an encrusted ureteral stent was removed using flexible URS with a Ho: YAG laser. Flexible URS has particular advantages for treating encrusted ureteral stents at the proximal end of the loop. This procedure is less invasive and is thus considered to be suitable for encrusted ureteral stents in the renal pelvis.
